# Deciphering the Effect of Postharvest 1-MCP Treatment Coupled with Low-Temperature Storage on the Physiological Activities and Edible Quality of Melon

**DOI:** 10.3390/plants14040586

**Published:** 2025-02-14

**Authors:** Haofei Wang, Zhiyi Yang, Sikandar Amanullah, Huilin Wang, Bin Liu, Shi Liu, Tiantian Yang, Chaonan Wang

**Affiliations:** 1 College of Horticulture, Xinjiang Agricultural University, Urumqi 830052, China; wanghaofei181@163.com (H.W.); yangzhiyi1996@163.com (Z.Y.); wanghuilin@126.com (H.W.); 2 Xinjiang Special Melon and Fruit Variety Improvement and Logistics Transportation Joint Research Center, Xinjiang Agricultural University, Urumqi 830052, China; 3 Department of Horticultural Science, North Carolina State University, Mountain Horticultural Crops Research and Extension Center, 455 Research Drive, Mills River, NC 28759, USA; sikandaraman@yahoo.com; 4 Hami Melon Research Center, Xinjiang Academy of Agricultural Sciences, Urumqi 830091, China; liubincau@163.com; 5 College of Horticulture, Northeast Agricultural University, Harbin 150030, China; shiliu@neau.edu.cn

**Keywords:** 1-MCP, melon, postharvest, quality, physiology, shelf life

## Abstract

Fruits are an important source of a healthy diet due to their essential nutrients for daily intake. Melon is known as a significant fruit crop of the Cucurbitaceae family based on its various dietary benefits, but its shelf life needs to be maintained for long-term usage. 1-Methylcyclopropene (1-MCP) is a cyclopropene-derived synthetic plant growth regulator (PGR) that is used for significantly delaying the ripening process and maintaining the shelf life of climacteric fruits during storage. In this study, freshly harvested melon fruits were fumigated with various concentrations (1.0 µL·L^−1^, 2.0 µL·L^−1^, and 3.0 µL·L^−1^) of 1-MCP treatment for 12 h (h) and stored at low temperature (8 ± 1 °C) for 30 days (d). The obtained results showed that 1-MCP fumigation coupled with low-temperature treatment maintains the postharvest shelf life of melon fruit. It was noticed that the increase in color hue (a* (red/green), b* (blue/yellow), L* (lightness)) was slowed down and the external fresh color was effectively maintained. At the same time, the firmness, soluble solids, titratable acids (TAs), and vitamin C (VC) content seemed to be maintained at a high level; weight loss and cell permeability were reduced; respiratory intensity and ethylene emission were inhibited; and the accumulation of superoxide anions and malondialdehyde (MDA) was also reduced. In addition, an upsurge in the activities of superoxide dismutase (SOD), catalase (CAT), peroxidase (POD), and ascorbate peroxidase (APX) was noticed in melon fruits under the combined treatment of 1-MCP and low-temperature storage as compared with the control group (CK, without treatment), indicating that 1-MCP treatment can effectively enhance the antioxidant metabolism of melon fruits during storage. Overall, we can recommend that the 3.0 µL·L^−1^ concentration of 1-MCP had the best effect on maintaining the internal and external quality of sweet melon fruit during storage.

## 1. Introduction

Fruits are classified into climacteric and non-climacteric types based on their versatile respiration patterns throughout the ripening phase. The climacteric fruits can continue to ripen after harvesting; however, they need to retain their freshness and extend shelf life against decreasing the postharvest economic loss caused by high metabolic activities. The upsurge in the respiration rate of climacteric fruits is commonly linked to the autocatalytic activity of ethylene (plant growth regulator), which regulates numerous ripening phases as well as sensory quality [1,2].

Melon (*Cucumis melo* L.) is one of the most popular horticultural fruit crops in the world due to its refreshing flesh with low calories and high nutritional value [3]. It has gradually moved away from seasonality to become a year-round favorite fruit. However, the freshly harvested melons need to be handled with proper care to avoid significant postharvest losses and to maintain the fresh quality, nutritional contents, taste, and sensory characteristics [4]. Melon fruit is highly respiratory at room temperature and ultimately disposed to post-ripening softening, water loss, skin shriveling, nutrient loss, and spoilage of edible quality. The advanced storage and transportation technologies have enabled the preservation of melons in optimal condition for extended periods and their rapid global transportation. Therefore, melons urgently need green and efficient storage technology to maintain their fresh quality and commercial value [5]. 

An optimal technique of postharvest handling comprises suitable preservation temperature, proper storage time, average humidity, modified atmosphere packaging, and physical or chemical treatments that can slow down the biological activities caused by senescence and maturation, as well as reduce or inhibit the development of physiological disorders [1,2]. 1-Methylcyclopropene (1-MCP) is considered a competitive inhibitor of ethylene biosynthesis. It has the advantages of non-toxicity, good chemical stability, easy synthesis, and low concentration [6]. In recent years, many researchers and scholars have evaluated the beneficial efficacy of exogenous 1-MCP treatment to delay the ripening and senescence and achieve the extended shelf life of fruits and vegetables. Nowadays, this technique is being used as commercial application due to the absence of harmful substances and eco-friendly advantages of safety, preservation, and storage. The previous studies have shown that 1-MCP application can maintain the postharvest quality by enhancing the obvious reactive oxygen species (ROS), reducing intracellular ROS production and accumulation, alleviating oxidative damage, and delaying the aging of fruits, e.g., pear [7], apple [8], kiwi [9,10], melon [4,11], western bracken fern [12], banana [13], and peach [14]. Some research in postharvest handling of fruits and vegetables has shown that ethylene-sensitive varieties receive more benefits of 1-MCP treatment by inducing the significant retardation of physicochemical parameters, reducing respiratory activity and nutrient loss, delaying maturation and ethylene production, maintaining high biological activity, and preserving their flavor and edible quality, e.g., sweet melons [4], tomatoes [15], bitter melons [16], mustard greens [17], and cabbage [18].

To date, several good varieties with improved edible and sensory properties of melon have been produced in China. Melon variety (Xizhou Mi, No. 25) is a member of the *Cucumis* genus of the Cucurbitaceae family [19] and originated from the Xinjiang region of China. This variety is an outstanding result of the improvement of local melon varieties and has been widely planted and consumed in other regions of China [20], due to its richness in vitamin C (VC), beta-carotene, polyphenol antioxidants, and potassium elements for the body. However, there is a dire need to maintain the shelf life of this variety to make it available at a commercial scale during the off-season. Many studies have shown that using low temperature or 1-MCP treatment alone can achieve the goal of extending the shelf life of sweet melons, but there is limited research on deciphering the effects of combined usage of low temperature and 1-MCP treatment on the physiological activities and nutritional quality of sweet melons. 

Herein, in order to provide a theoretical basis for the long-term storage and preservation of melon, we tried to check the effect of the exogenous application of 1-MCP treatment on the postharvest quality, nutritional components, and antioxidant activity of melon variety (Xizhou Mi, No. 25) in a low-temperature storage environment.

## 2. Results

### 2.1. Effect of 1-MCP Treatment on the Color Change in Melon Peel

**Figure 1:**
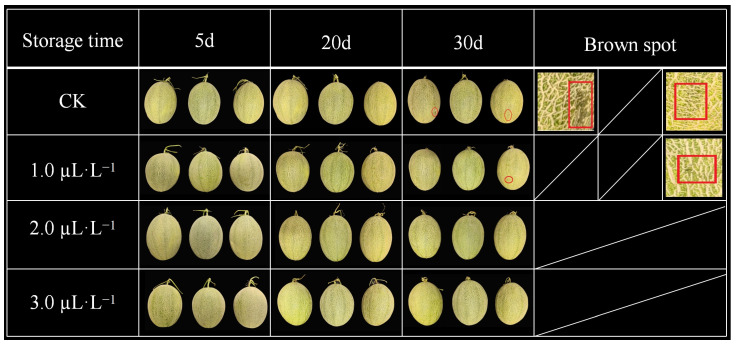
The visual fruit appearance of melon variety (Xizhou Mi, No. 25) treated with different concentrations of 1-MCP and low-temperature storage (8 ± 0.5 °C). The small red circles are indicating the signs of decay under different treatments. In the expanded figure, melon fruit skin showed brown spots with signs of rotting. The stalks of melon fruits showed variation in physical morphology due to water loss throughout the storage time.

The color change in fruit peel is an important indicator for evaluating fruit maturity and quality. The color hue (L* value, a* value, and c* value) is one of the most commonly used methods for determining color changes. Herein, the glossiness of the melon peel became darker, the green area of the peel became lighter, and the color turned yellow after 1-MCP treatment. The detailed visual observation of melon color and shine during storage revealed that the color and shine changes were not obvious at 10–15 days of storage, but the shine began to darken significantly, and the skin color became lighter and yellowish at the 20th day of storage. However, on the 30th day of storage, brown spots appeared in the 1 µL·L^−^^1^ of the 1-MP-treated group as compared to the control, which showed signs of rotting (Figure 1).

The L* value represents the significant brightness value of the outer skin, because a higher value depicts better glossiness, and a smaller value shows decreased glossiness; however, the L* value gradually decreased during the entire storage period (Table 1), indicating a darkening of epidermal shine. On the 20th to 30th day of the storage interval, the L* value of the treatment group with the 3.0 µL·L^−1^ of 1-MCP was significantly higher than that of the control group, and the overall L* value was slowly decreased in 3.0 µL·L^−1^ of 1-MCP treatment.

The value of a* represents the change between red and green; negative values represent green, positive values represent red, and the larger the absolute value, the darker the color. As the storage time increased, the overall absolute value of a* of each group gradually decreased, indicating that the melon peel color became lighter (Table 1). The 1-MCP-treated group showed significantly higher color than the control group (which showed rapid color changes) during the 10–30-day storage interval. The absolute a* value was noticed to be highest in the 3.0 µL·L^−1^ of the 1-MCP-treated group. 

In addition, the b* value represents the change between yellow and blue; negative values represent blue; and positive values represent yellow. It was noticed that the larger the absolute value, the darker the color of melon fruit peel. As the storage time increased, the absolute value of the total b* value of each group gradually increased, indicating that the color of the melon peel gradually turns yellow (Table 1), and the 3.0 µL·L^−1^ of 1-MCP treatment positively and significantly maintained the higher b* value than that of the lowered value observed in the control group, respectively.

### 2.2. Effect of 1-MCP and Low-Temperature Treatment on the Skin Firmness, Total Soluble Solids, Titratable Acidity, and Ascorbic Acid Content of Melon

**Figure 2:**
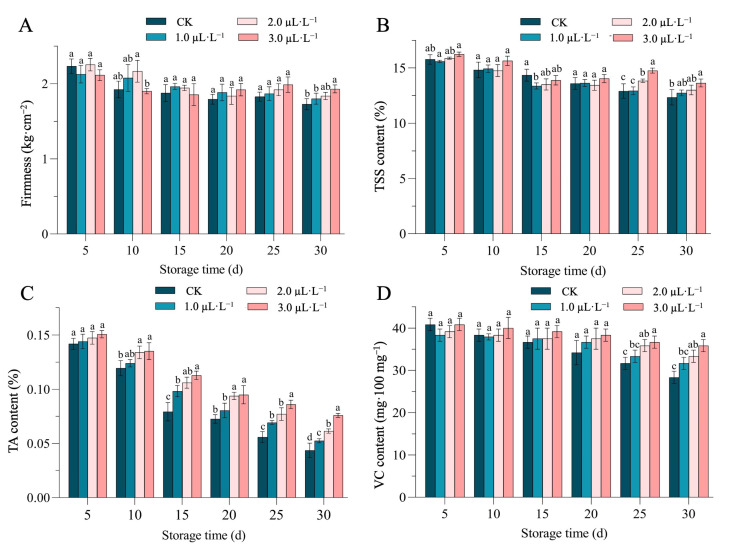
Effect of 1-MCP and low-temperature treatment on (**A**) firmness, (**B**) TSS, (**C**) TA, and (**D**) VC of melon fruits. Different letters indicate significant difference between treatments at each time point (*p* < 0.05) based on Duncan’s multiple range (DMR) test, respectively.

Fruit firmness is an indicator that reflects the quality changes and softening degree of fruits during postharvest storage. In this experiment, the overall fruit firmness showed a decreasing trend with the increase in storage time (Figure 2A), and the fruits with control (CK) treatment showed the most significant decrease on the 5th-10th day due to obvious nutritional depletion. The fruit firmness was significantly higher in the fruit groups treated with 1.0 µL·L^−1^ and 3.0 µL·L^−1^ concentrations of the 1-MCP than that of the control group on the 30th day, and there was no significant difference compared to the 2.0 µL·L^−1^ of the 1-MCP group (*p* < 0.05), respectively. However, the fruit group treated with the 3.0 µL·L^−1^ concentration of the 1-MCP had the best effect in inhibiting the decrease in fruit firmness.

TSS content is an important quality factor that affects the commercial worth. Herein, we noticed that TSS content of cantaloupe fruits showed an overall slightly decreasing trend (Figure 2B), and each treatment had no significant effect on the total soluble sugar content of cantaloupe fruits stored for 5 to 20 days. However, the fruit samples treated with 2.0 µL·L^−1^ and 3.0 µL·L^−1^ of 1-MCP exhibited significantly higher TSS content than the CK and other 1-MCP groups on the 25th day of storage. However, the fruit group treated with the 3.0 µL·L^−1^ concentration of 1-MCP showed significantly higher content than the CK at 30 days of storage. 

On the other hand, TA and VC content are also important components of the flavor and nutrition of sweet melon fruits (Figure 2C,D). TA content showed a positively higher trend in the fruit group treated with 2.0 µL·L^−1^ and 3.0 µL·L^−1^ concentrations of 1-MCP than that in the control (CK) group on the 15th and 25th days of storage. However, the 3.0 µL·L^−1^ of 1-MCP treatment showed a significantly higher value than the control group at 30 days of storage (Figure 2C). 

The content of VC continued to decrease with the extension of storage time, and there was no significant difference between the VC content of the treatment groups from day 5 to day 20. However, the advantage of 3.0 µL·L^−1^ of 1-MCP concentration gradually emerged from day 25 to day 30. The VC content of the fruit group treated with the 3.0 µL·L^−1^ concentration of 1-MCP was significantly higher than the group treated with the 1.0 µL·L^−1^ concentration of 1-MPC and the CK group; however, no significant difference was noticed compared to the group treated with 2.0 µL·L^−1^ of 1-MCP treatment (Figure 2D).

### 2.3. Effect of 1-MCP Treatment on Weight Loss Rate, Cell Membrane Permeability, Respiration Rate, Ethylene Production, O_2_^−^ Generation Rate, and Malondialdehyde Content of Melon

**Figure 3:**
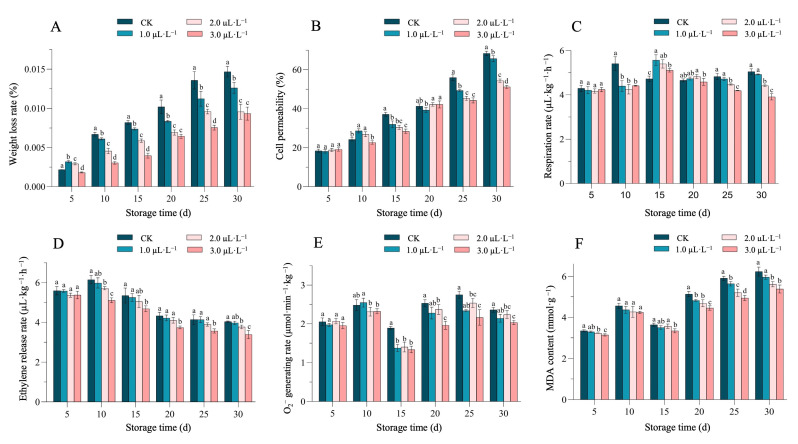
Effect of 1-MCP and low-temperature treatment on the (**A**) weight loss% rate, (**B**) cell membrane permeability, (**C**) respiration rate, (**D**) ethylene production, (**E**) O_2_^−^ generation rate, and (**F**) malondialdehyde content in melon fruits. Different letters indicate a significant difference between treatments at each time point (*p* < 0.05) based on Duncan’s multiple range (DMR) test, respectively.

The weight loss rate (%) and cell membrane permeability are key indicators for the storage quality and market value of sweet melons. The 1-MCP fumigation significantly reduced the weight loss rate of cantaloupe after 10 days of storage as compared with the control group. The rate of weight loss was 0.015%, 0.013%, 0.010%, and 0.009% in the stored control and 1.0 µL·L^−1^, 2.0 µL·L^−^^1^, and 3.0 µL·L^−1^ treatment groups on the 30th day of storage, respectively (Figure 3A).

During the entire storage period, the cell permeability (%) of the fruit showed an overall upward trend. On the 10th day of storage, the cell permeability of the 1.0 µL·L^−1^ and 2.0 µL·L^−1^ of 1-MCP-treated groups was significantly higher. However, the 1-MCP fumigation significantly reduced fruit permeability after 25 days of storage, with each treatment group reaching its peak on the 30^th^ day. The cell permeability of the control groups and the 1.0 µL·L^−1^, 2 µL·L^−1^, and 3.0 µL·L^−1^ treatment groups had significant values of 68.32%, 65.63%, 54.35%, and 51.17%, respectively (Figure 3B).

The measurements of the respiratory intensity (mL·kg-1·h^−^^1^) and ethylene release rate directly affect the climacteric behavior of melon fruits. Herein, we observed the fluctuations in these traits of cantaloupe throughout the entire storage period (Figure 3C and Figure 3D), respectively. The sample group treated with 1-MCP concentration (3.0 µL·L^−1^) showed an overall upward and then gradual downward trend; however, the respiratory rate of the control group reached its peak at 5.41 µL·kg^−1^·h^−1^ on the 10th day of storage, while the 1-MCP-treated group delayed the peak by 5 days. On the 25th and 30th days of storage, the respiration rates of the sample treated with 2.0 µL·L^−1^ and 3.0 µL·L^−1^ of 1-MCP showed a significantly lowered trend than the CK (Figure 3C).

In the determination of ethylene content, it was found that the 1-MCP treatment group showed an overall decreasing trend. On the 10th day of storage, the ethylene content of all treatments reached its maximum value, and the ethylene release of 3.0 µL·L^−1^ was 16.92% lower than that of the CK. Afterwards, the ethylene release in the 3.0 µL·L^−1^ 1-MCP treatment group was significantly lower than that in other treatment groups. The 3.0 µL·L^−1^ 1-MCP fumigation inhibited the respiration of sweet melons and reduced the production of ethylene (Figure 3D).

As an important indicator of intracellular reactive oxygen species (ROS), the excessive accumulation of O_2_^−^ can cause irreversible damage to cells. In our experiment, the O_2_^−^ content showed an overall fluctuation trend during storage, and the 1-MCP treatment inhibited O_2_^−^ in treated groups after 15 days of storage; however, the accumulation of O_2_^−^ in melons treated with 1-MCP was significantly lower than that in the control group, and the lowest peak was observed. Further, the rate of O_2_^−^ production in the samples treated with 2.0 µL·L^−1^ and 3.0 µL·L^−1^ of 1-MCP was significantly lower than the CK, while the 3 µL·L^−1^ 1-MCP was more effective in reducing O_2_^−^ during storage for 20–30 days (Figure 3E).

MDA is the final product of lipid peroxidation, and its accumulation indicates the presence of oxidative free radicals in cells, which disrupt cell membrane structure and interfere with normal physiological metabolism. In this postharvest study, the obtained results showed that the group samples treated with 3.0 µL·L^−1^ of 1-MCP concentration showed significantly lower malondialdehyde content than that in the control group and the other two 1-MCP treatment groups (Figure 3F).

### 2.4. Effect of 1-MCP Treatment on Activities of Superoxide Dismutase (SOD), Catalase (CAT), Peroxidase (POD), and Ascorbate Peroxidase (APX) in Melon

**Figure 4:**
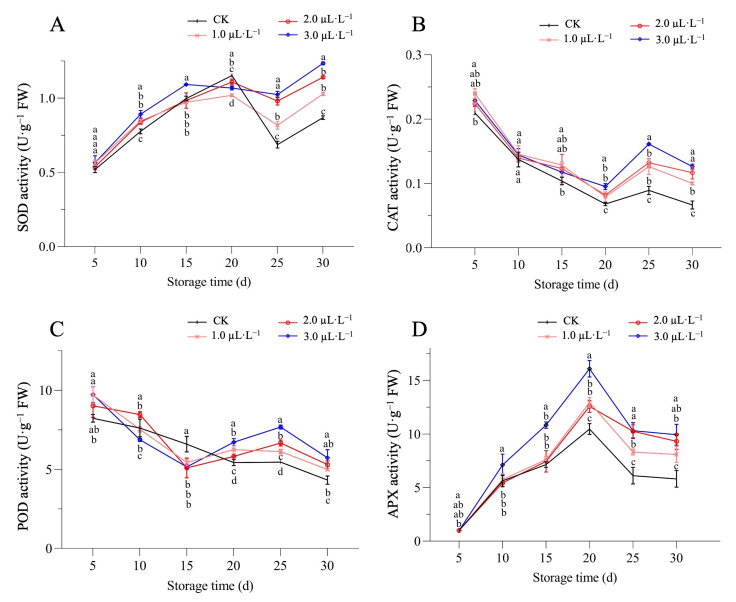
Effect of 1-MCP and low-temperature treatment on (**A**) SOD, (**B**) CAT, (**C**) POD, and (**D**) APX activities in melon fruits. Different statistical letters indicate significant difference between treatments at each time point (*p* < 0.05) based on Duncan’s multiple range (DMR) test, respectively.

The obtained results revealed that SOD content (U·g·min) in melon fruits had an overall trend of first increasing, then decreasing, and then increasing again. The SOD activity in the samples treated with 3.0 µL·L^−1^ of 1-MCP concentration was significantly higher than the other groups on the 15th day of storage. However, the CK was significantly higher than the 1-MCP treatment group on the 20th day of storage, and the decline rate of the 1-MCP treatment group was slower, and the SOD activity was higher than that of the control group on the 20th to 25th day of storage (Figure 4A). 

The CAT (U·g^−^^1^) and POD (U·g^−^^1^) activity showed a similar trend of first decreasing, then increasing, and then again decreasing during the overall postharvest storage process. The activity of catalase in melon flesh was relatively high, while the stimulation of CAT activity by treatment of 3.0 µL·L^−1^ of 1-MCP concentration was more significant in the later stage of storage (20–30 days). The CAT activity of melon treated with different concentrations of 1-MCP was significantly higher than that of the CK. In addition, samples treated with 3.0 µL·L^−1^ of 1-MCP concentration showed a rapid increase in catalase activity after 20–25 days of storage (Figure 4B). However, the POD activity of the control group was significantly higher than the other 1-MCP groups after 15 days of storage, but the control group showed a decreasing trend in the POD activity, while the 1-MCP treatment group showed an increasing trend after 15–20 days of storage (Figure 4C).

In addition, the APX content (U·g^−^^1^) remained significantly higher in the sample groups treated with 3.0 µL·L^−1^ of 1-MCP concentration than in other 1-MCP treatment groups and the control group during 10–20 days. However, peaks were observed in all treated groups on the 20th day of storage, and the highest peak was observed in the 3.0 µL·L^−1^ treatment group, reaching up to 16.09 U·g^−^^1^ and showing a decreasing trend in the later stages (Figure 4D).

## 3. Discussion

Fresh horticultural crops are being produced at a large scale in the world, but approximately 25–30% postharvest losses take place every year due to non-efficient harvesting methods and improper postharvest handling. Melon is known as a pivotal fruit crop due to its beneficial nutrients, but due to various uncertain factors such as seasonality, growth conditions, perishability, and postharvest diseases, the production of melon fruits is prone to losses after harvesting, which can affect their physiological traits and nutritional quality [21]. However, a lot of non-destructive and effective techniques have been introduced for handling the postharvest losses and maintaining the shelf life of fruits and vegetables by slowing down the respiration rate, delaying senescence, and decreasing the tissue breakdown [14].

The respiratory intensity of fruits and vegetables is closely related to the external environmental temperature. The low storage temperature triggers the reduction in respiratory intensity of fruits and vegetables. It was reported that 1-MCP treatment combined with low temperature played a functional role as a dual inhibitory effect for delaying the postharvest ripening process of melon due to ethylene emission, and fruits can be stored for up to 30 days [22]. This result is consistent with previous reports on ‘Galia’ melons, but their use of 1-MCP had no effect on quality loss, total titratable acidity, soluble solids, or total soluble sugars [23,24]. There may be potential differences in the effects of 1-MCP on different melon varieties. Similarly, treatment with 1-MCP reduced the soluble solids content of ‘orange flesh’ and ‘Charantais’ melons, while treatment with 1-MCP had no effect on ‘Galia’ [25]. The effects of 0.1, 1.0, and 5.0 µL·L^−1^ of 1-MCP treatment were studied at room temperature for 24 h, and the comprehensive effect of 5 µL·L^−1^ of 1-MCP concentration on the storage of melon was more significant than that of the control group. This is consistent with the obtained findings in our experiment where the fruit peel of melon exhibited brown spots and necrosis as the shelf life of melon was extended, but the low-temperature storage and 1-MCP treatment maintained the visual appearance and quality of melon, as the significant effects of various concentrations can be noticed (Figure 1).

In recent years, the 1-MCP application has emerged as a postharvest storage treatment due to its effective role as an eco-friendly and competitive inhibitor of ethylene and for preventing postharvest diseases [2,26]. Recently, the 1-MCP treatment was widely applied for a few cases of maintaining the shelf life of various fruits, e.g., kiwifruit [27], mango [28], and blueberry [29,30]. It was found that 1-MCP treatment can better maintain the hardness of ‘Hayward’ and ‘Qi Hong’ kiwifruit and delay the increase in external color hues (L*, a*, and b*) [9]. Further, it was found that 1-MCP treatment inhibited the softening and weight loss of figs and delayed the decrease in total soluble solids, titratable acidity, and VC content [31]. In this study, we similarly investigated the effect of different concentrations of 1-MCP combined with low-temperature treatment on the storage quality of melon. The acquired results showed that different concentrations of 1-MCP treatment could inhibit color degradation (Table 1), by maintaining the hardness/firmness, high titratable acidity, TSS contents, and VC content levels (Figure 2), as well as maintained the weight loss rate%, cell membrane permeability, respiration rate, ethylene production, O_2_^−^ generation rate, and malondialdehyde content in melon fruits (Figure 3), but the storage effect was the best under 3.0 µL·L^−1^ of 1-MCP treatment. These significant results are consistent with the previous results of postharvest studies in kiwi [9] and melon [21] where the shelf life of fruits was maintained for the next several days compared to the expected storage time, by inhibiting the metabolic activities, respiration rate, and gaseous exchange rate.

The changes in relative conductivity and MDA content reflect the degree of damage to the cell membrane. When the cell membrane is damaged externally, there is an imbalance in osmotic pressure between the inside and outside of the cell, leading to the leakage of intracellular substances and an increase in electrical conductivity [12]. MDA is one of the final products of lipid peroxidation in the cell membrane, and its accumulation directly reflects the fluctuated degree of membrane lipid peroxidation, indirectly indicating the damage status of cell membrane structure [32]. The elevated levels of superoxide anions (a type of reactive oxygen species) can exacerbate oxidative stress levels in cells, further exacerbating damage [33]. Hu et al. [34] evaluated the effectiveness of 1-MCP in slowing down the rate of O_2_^−^ production and reducing postharvest MDA accumulation in cabbage. 

Meanwhile, SOD, CAT, POD, and APX are known to be important antioxidant enzymes for eliminating ROS, and their activity is closely related to the homeostasis of ROS in fruits [2,35,36,37]. SOD can disproportionate O_2_^−^ production to H_2_O_2_ and O_2_ through the disproportionation reaction, while H_2_O_2_ can be further decomposed into H_2_O and O_2_ by CAT and APX enzymes in concert, where POD enzymes catalyze the decomposition of H_2_O_2_, and at the same time use the oxidative properties of hydrogen peroxide to oxidize the reductant RH_2_, forming H_2_O and the oxidation product “R” [38]. The activity and interaction between these antioxidant enzymes have been shown to be related to lipid peroxidation and aging delay mechanisms in many horticultural crops. Similar effects were also found in apples [39] and peaches [18], where treatment with 3.0 µL·L^−1^ of 1-MCP concentration showed the best effects of increased antioxidant enzyme activity and extended shelf life. These results are in line with the obtained results of our experimental study, which indicated that 1-MCP treatment could effectively enhance the activity of antioxidant enzymes as compared with the control group (Figure 4), suggesting that 1-MCP could reduce ROS accumulation by increasing the activity of ROS-metabolizing enzymes and enhancing the antioxidant capacity of melons.

## 4. Materials and Methods

### 4.1. Sample Processing

In this study, we selected a climacteric type of melon variety (Xizhou Mi, No. 25) as an experimental material for studying the postharvest physiological traits. The fruits were freshly harvested from the large greenhouse production base of the Grape and Melon Research Institute in Shanshan County, Turpan City, Xinjiang Uygur Autonomous Region of China. We selected ripened fruits with uniform size, free of disease, pests, and mechanical damage, and immediately transported them back to the postharvest storage laboratory of fruit and vegetables of the Grape and Melon Research Institute. The various concentrations (1.0 µL·L^−1^ (0.33 g powder of 1-MCP), 2.0 µL·L^−1^ (0.66 g powder of 1-MCP), and 3.0 µL·L^−1^ (0.99 g powder of 1-MCP)) were formulated and applied on melon fruits using a triplicate form in each treatment group, respectively.

### 4.2. Experimental Design

The freshly harvested melons were shifted into the laboratory, and field heat was removed by keeping them in a room in an ambient environment (24 ± 1 °C temperature and ~70% relative humidity) for one night. Then, fruits were randomly divided into four treatment groups (T0 (CK, control), T1 (1.0 µL·L−1), T2 (2.0 µL·L−1), and T3 (3.0 µL·L−1)), with 18 fruits in each treatment group, followed by three pseudo-replications of each treatment, respectively. A total of six fruits in each treatment were placed in cardboard boxes (43.75 L capacity) and fumigated at an ambient temperature of 24 ± 1 °C for 12 h. The 1-MCP treatment bags were then immersed in deionized water to moisten the bags and quickly placed in a box made of polyethylene (PE) film (0.1 mm thick), placed in a sealed box, transferred to refrigeration (8 ± 0.5 °C), and stored at a relative humidity of 75% to 85%. The melons of the control treatment group were sealed in a container at the same temperature for 12 h in the absence of 1-MCP, placed in cold storage (8 ± 0.5 °C), and physiological indicators, including the ethylene production and respiration rate, were measured at intervals of 5 days each, with three biological replicates per group. In addition, three pieces of melon flesh along with peel were taken from each treatment; the peeled flesh was cut into small pieces of 0.5 cubic centimeters (cm^3^, 1 cm × 1 cm × 1 cm), rapidly frozen with liquid nitrogen, and stored at −80 °C for the determination of physiological indicators.

### 4.3. Determination of Chromaticity and Firmness of Fruits

We checked the peel color of melon fruit from 10 points along the skin’s equator, by repeating the process three times for each group, and color hue values (a* (red/green), b* (blue/yellow), L* (lightness)) were estimated with an earlier-reported method [40]. Fruit firmness was measured using a hand-held penetrometer (GY-1, Firmness Tester, Hangzhou, China) using some modifications of an earlier-reported method described in [11]. In short, a total of three melon fruits from each treatment/group were cut along the transverse midline; 10 points were selected evenly at the flesh depth of 2.0 cm from the peel surface, the measuring head was pressed vertically and evenly (0.8 cm × 0.8 cm and 2 cm deep), and the final data were recorded, respectively.

### 4.4. Measurement of Physiological and Biochemical Indicators

Total soluble solids (TSSs) were determined according to the previous method of Zhang et al. [11]. Titratable acidity (TA) was measured by a titration method, using 0.01 mol/L NaOH and phenolphthalein as the color indicator method of Ma et al. [41]. Ascorbic acid (AsA) content was determined from the flesh extract using the 2,6-dichlorophenol-indophenol titration method [42]. The cell membrane permeability was measured using slight modifications in the method proposed by Lin et al. [43]. The malondialdehyde (MDA) content was measured using the method described by Duan et al. [44] and modified. O_2_^−^ production rates were determined according to Yang et al. [45]. 

The postharvest respiration rate of fruit was determined based on the ethylene emission rate using the method described by Li et al. [46]. The improved guaiacol method was used to determine POD activity based on the reported method of Yang et al. [45], CAT activity was measured according to Dhindsa et al. [47], and SOD activity was also determined by measuring its ability to inhibit the photo-reduction of nitroblue tetrazole (NBT) [47]. The activity of APX was determined using slight modification in the method of Tewari et al. [48].

### 4.5. Statistical Analysis

All the physiological and quality parameters were checked at different days (5 d, 10 d, 15 d, 20 d, 25 d, and 30 d), and data were recorded from three pseudo-replications of each treatment. The standard statistics (mean ± standard deviation) and significant differences in each treatment were checked at *p* < 0.05 based on Duncan’s multiple range and the one-way analysis of variance test using SPSS software (version 22.0).

## 5. Conclusions

As the crux, we conclude that 1-MCP application coupled with low-temperature treatment showed a positive and significant effect on maintaining the shelf life of melon fruit. The treated melons exhibited good visual appearance and quality characteristics by effectively slowing down the degradation of external color and maintaining the skin firmness, total soluble solids, titratable acid, and VC content, as well as inhibiting respiratory intensity, reducing ethylene emission, and delaying fruit decay and aging. Overall, the 3.0 µL·L^−1^ concentration of 1-MCP treatment coupled with low temperature was exhibited as the best combination, which showed an optimistic effect on maintaining the edible quality and regulation of enzyme activity metabolism and the preservation and storage of melon variety (Xizhou Mi 25).

## Figures and Tables

**Table 1:** Effect of 1-MCP and low temperature on changes in color hue values (a* (red/green), b* (blue/yellow), L* (lightness)) during storage of melons, respectively.

Color Hues	Treatment	Storage Time (Days, d)					
		5 d	10 d	15 d	20 d	25 d	30 d
L* value	CK	55.38 ± 0.68 ^a^	53.25 ± 1.09 ^a^	51.97 ± 0.71 ^a^	50.52 ± 0.67 ^a^	49.83 ± 0.47 ^a^	48.83 ± 1.73 ^a^
	1.0 µL/L	55.60 ± 1.14 ^a^	53.41 ± 1.10 ^a^	52.10 ± 1.06 ^a^	51.38 ± 0.38 ^ab^	50.72 ± 0.64 ^ab^	49.61 ± 0.70 ^ab^
	2.0 µL/L	55.41 ± 1.12 ^a^	53.53 ± 1.28 ^a^	52.56 ± 0.74 ^a^	51.50 ± 1.09 ^ab^	51.12 ± 1.08 ^ab^	50.37 ± 0.88 ^ab^
	3.0 µL/L	55.54 ± 0.77 ^a^	53.89 ± 0.18 ^a^	53.13 ± 0.77 ^a^	52.72 ± 1.18 ^b^	51.60 ± 0.61 ^b^	51.08 ± 0.50 ^b^
a* value	CK	−2.57 ± 0.11 ^a^	−1.76 ± 0.06 ^a^	−1.69 ± 0.08 ^a^	−1.48 ± 0.08 ^a^	−1.03 ± 0.11 ^a^	−0.83 ± 0.08 ^a^
	1.0 µL/L	−2.57 ± 0.11 ^a^	−2.53 ± 0.23 ^b^	−2.32 ± 0.10 ^b^	−1.79 ± 0.05 ^b^	−1.65 ± 0.20 ^b^	−1.32 ± 0.11 ^b^
	2.0 µL/L	−2.95 ± 0.16 ^b^	−2.61 ± 0.05 ^b^	−2.32 ± 0.13 ^b^	−2.00 ± 0.15 ^c^	−1.79 ± 0.01 ^b^	−1.47 ± 0.11 ^b^
	3.0 µL/L	−2.93 ± 0.06 ^b^	−2.78 ± 0.13 ^b^	−2.46 ± 0.11 ^b^	−2.17 ± 0.12 ^c^	−1.78 ± 0.18 ^b^	−1.72 ± 0.11 ^c^
b* value	CK	17.80 ± 0.15 ^a^	19.33 ± 0.39 ^a^	19.97 ± 0.26 ^a^	20.56 ± 1.06 ^a^	21.86 ± 0.48 ^a^	23.15 ± 0.34 ^a^
	1.0 µL/L	17.42 ± 0.36 ^a^	18.50 ± 0.85 ^a^	19.70 ± 0.60 ^a^	20.36 ± 0.57 ^a^	21.55 ± 0.82 ^a^	22.1 ± 0.87 ^ab^
	2.0 µL/L	17.96 ± 0.63 ^a^	19.04 ± 1.12 ^a^	19.60 ± 0.91 ^a^	20.56 ± 0.84 ^a^	20.86 ± 1.06 ^a^	21.90 ± 0.68 ^ab^
	3.0 µL/L	17.58 ± 0.69 ^a^	18.63 ± 0.96 ^a^	18.70 ± 1.25 ^a^	19.82 ± 0.89 ^a^	20.46 ± 0.78 ^a^	21.35 ± 0.59 ^b^

Note: Different letters indicate a significant difference between treatments at each time point (*p* < 0.05) by Duncan’s multiple range (DMR) test, respectively.

## Data Availability

The original contributions presented in this study are included in the article. Further inquiries can be directed to the corresponding authors.
